# Weather or not—Global climate databases: Reliable on tropical mountains?

**DOI:** 10.1371/journal.pone.0299363

**Published:** 2024-03-13

**Authors:** Andreas Hemp, Judith Hemp

**Affiliations:** 1 Department of Plant Systematics, University of Bayreuth, Bayreuth, Germany; 2 Waldkunde-Institut Eberswalde, Eberswalde, Germany; 3 Friedrich-Alexander-Universität Erlangen-Nürnberg (FAU), Erlangen, Germany; City College of New York, UNITED STATES

## Abstract

Global, spatially interpolated climate datasets such as WorldClim and CHELSA, widely used in research, are based on station data, which are rare in tropical mountains. However, such biodiversity hotspots are of high ecological interest and require accurate data. Therefore, the quality of such gridded datasets needs to be assessed. This poses a kind of dilemma, as proving the reliability of these potentially weakly modelled data is usually not possible due to the lack of stations. Using a unique climate dataset with 170 stations, mainly from the montane and alpine zones of sixteen mountains in Tanzania including Kilimanjaro, we show that the accuracy of such datasets is very poor. Not only is the maximum amount of mean annual precipitation drastically underestimated (partly more than 50%), but also the elevation of the precipitation maximum deviates up to 850m. Our results show that, at least in tropical regions, they should be used with greater caution than before.

## Introduction

Global, spatially interpolated climate data on grids are widely used in scientific research in various disciplines, including ecology, biodiversity, conservation and climate change [1,2]. Such gridded datasets use global or continental networks of weather stations, e.g. GHCN-D [3] or HSM-SIEREM [4] for their modelling approaches. There are several gridded climate reanalysis datasets merging satellite and station data (e.g. CRU [5] or ERA5 [6]) with a rather coarse spatial resolution between 0.25 and 1 degree (which corresponds to about 28 to 100 km in the tropics). However, this resolution is much too coarse to capture orographic precipitation patterns in complex terrain [7,8]. Furthermore, for many questions and applications in environmental research and ecology, climate data with higher resolution is essential [2].

Databases such as WorldClim [9] (WC) provide free access to downscaled climate data with a spatial resolution of 30 arc seconds; this corresponds to about 0.86 km^2^ at the equator and is commonly referred to as "1-km" or "high" or "very high" resolution [2,7]. WC provides a range of climate variables derived from weather station records and other sources, such as temperature, precipitation, solar radiation, wind speed and humidity. WorldClim’s strengths include its comprehensive coverage, ease of use and availability of different spatial resolutions. CHELSA (Climatologies at High Resolution for the Earth’s Land Surface Area [10] (C) is another global climate database that focuses on providing such high-resolution climate data. It uses a combination of downscaled reanalysis data and satellite data to produce spatially explicit climate information.

Many studies published in high-impact journals, for example, have used WC data for their analyses. These studies often address topics such as species distribution, climate change impacts, ecosystem responses and environmental management strategies. In *PLOS ONE*, WC has been used in 920 studies since 2007, in *PNAS* in 131 studies since 2009, in *Science* in 49 studies since 2010, and in *Nature* in 44 studies since 2015.

However, many tropical regions lack consistent observations of even the most basic meteorological parameters such as temperature, humidity or precipitation. This holds particularly true for areas of complex terrain, such as the region of Kilimanjaro, Tanzania. On the other hand, it is exactly these regions that are of high ecological interest and value with regard to biodiversity and related ecosystem services, often termed ‘global biodiversity hot-spots’, where the accuracy of such data is an indispensable requirement for any meaningful analysis. Relying on results and conclusions based on above mentioned climate data sets modelled on a weak basis could be risky. Therefore, the quality of these tools needs to be assessed. However, proving the reliability of such data in remote tropical areas is mostly not possible due to the lack of stations, which poses a kind of dilemma.

In this study, we aim to verify the accuracy of WC and C–the two most frequently used data sets in biological research, with the highest available resolution—at 16 peaks on nine mountain ranges in Tanzania, with focus on Kilimanjaro, the highest mountain in Africa and the highest solitary mountain in the world using a unique climate dataset collected over the last 30 years mainly in the ecoclimatically most important montane and alpine zones.

**Fig 1:**
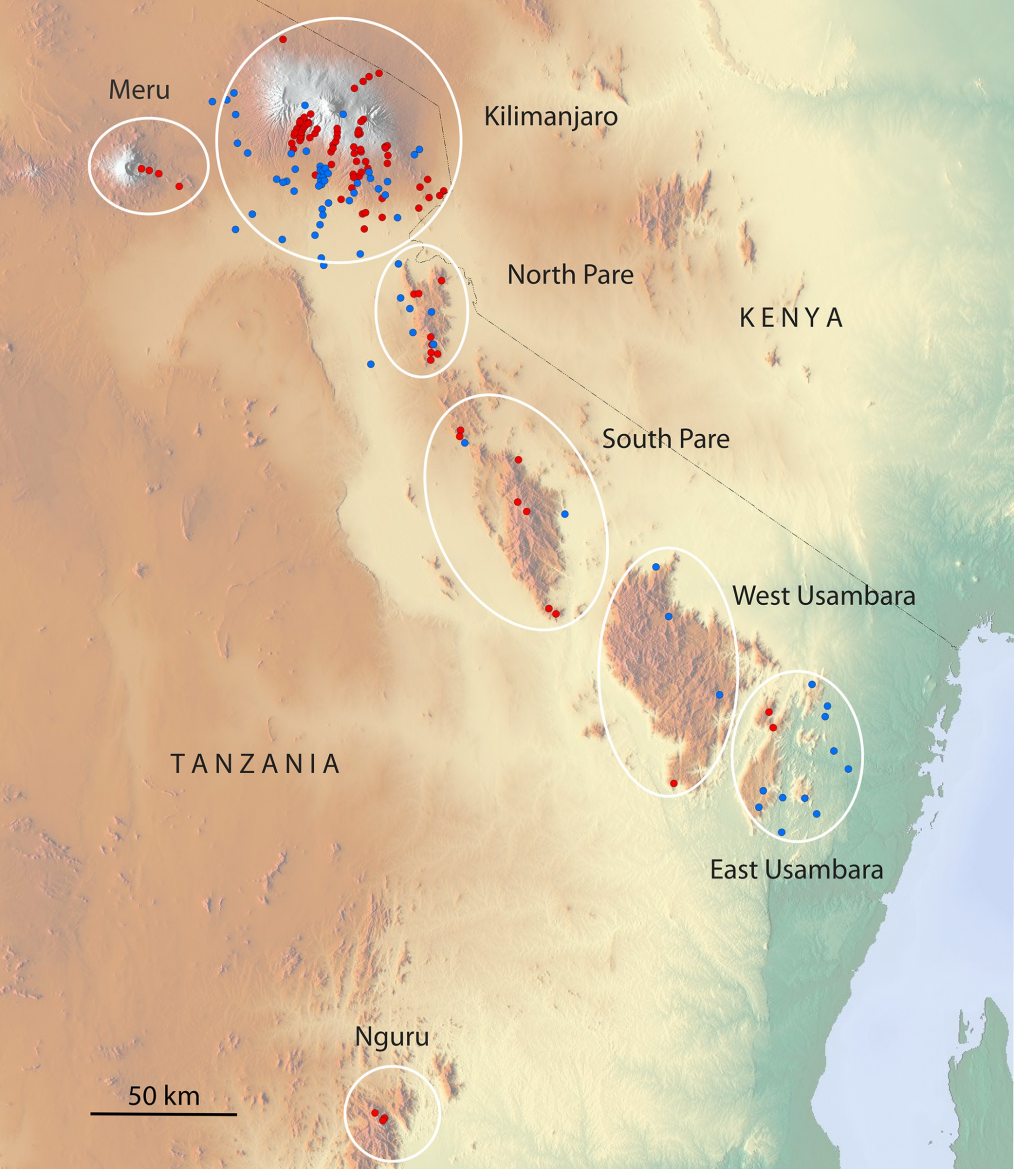
Location of used weather stations on Tanzanian mountains. Blue dots: Stations of various sources, red dots: Own stations. Map source: OpenStreetMap.

**Fig 2:**
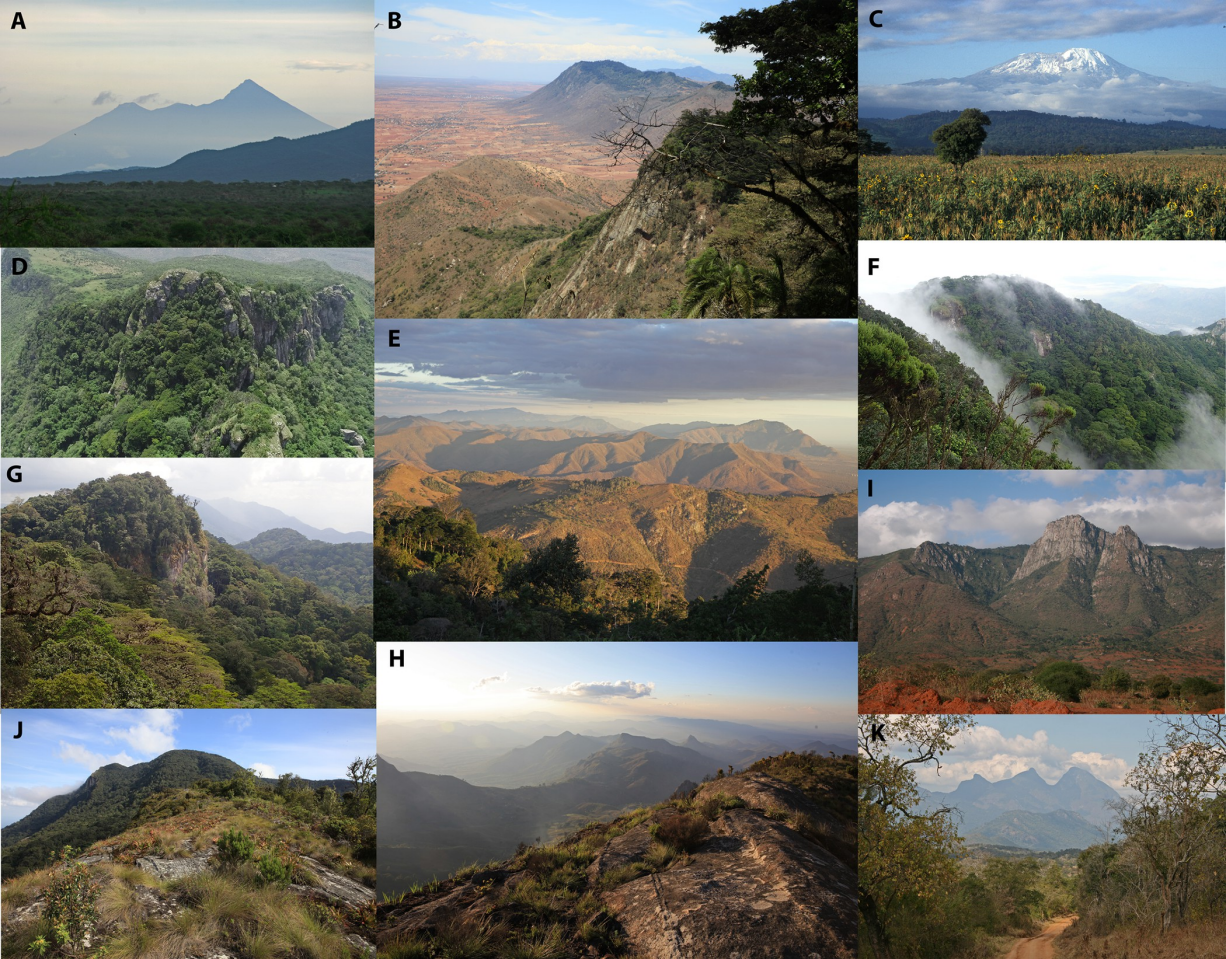
The studied Tanzanian mountain ranges. (A) Meru. (B) North Pare with Kindoroko in the background. (C) Kilimanajaro. (D) Vumari in South Pare. (E) South Pare with Shengena in the background. (F) Mwala in South Pare. (G) Nilo ind east Usambara. (H) Nguru. (I) West Usambara. (J) Makunguru in Nguru. (K) Kanga in Nguru. Whereas Kilimanjaro and Meru reach into the alpine zones, only the highest peaks of the other mountains are covered by remnants of montane (cloud) forest.

**Fig 3:**
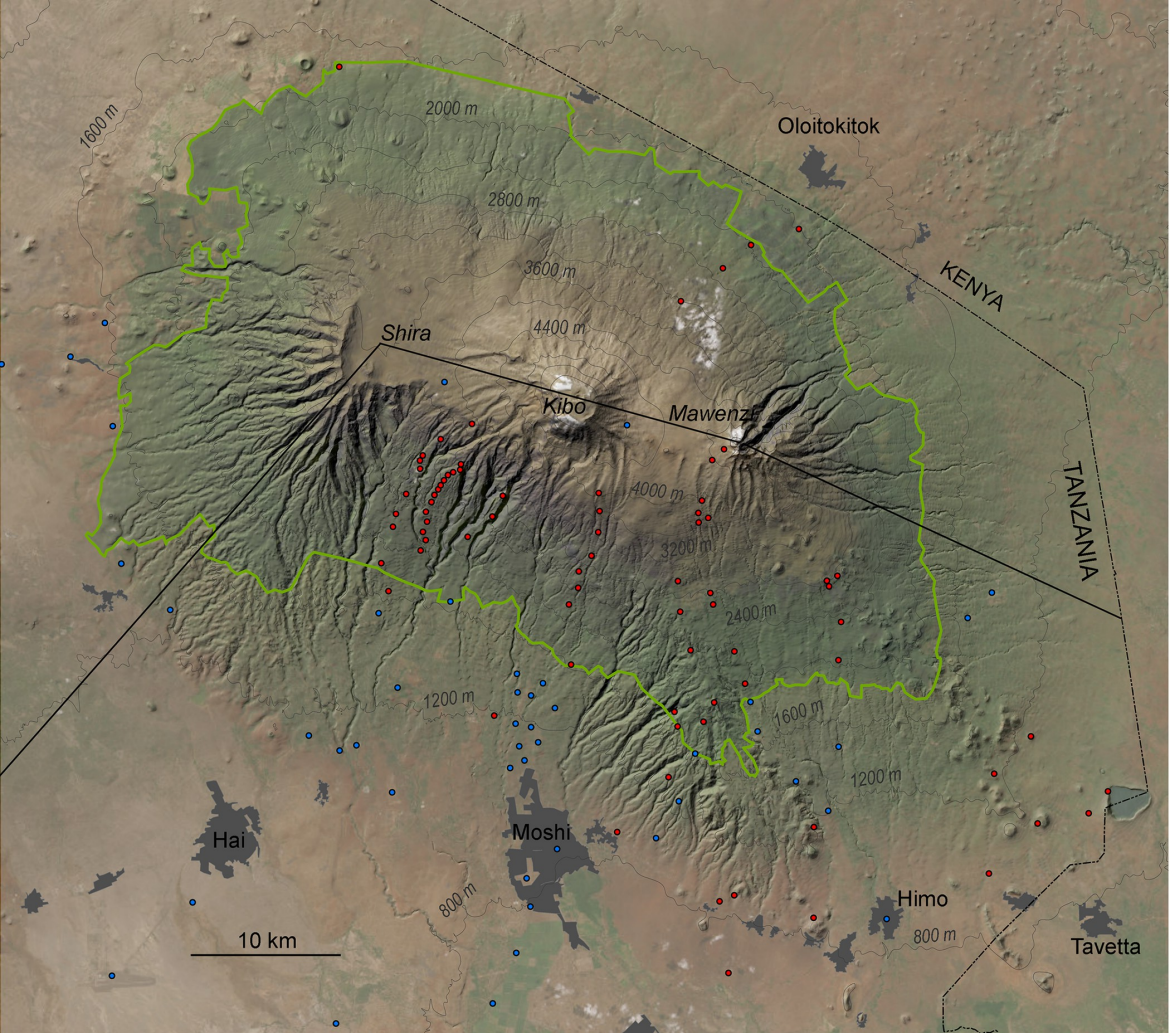
Location of used weather stations on Kilimanjaro. Blue dots: Stations of various sources, red dots: Own stations. Black line: Division of the northern and southern slope following [14]. Green line: Demarcation of Kilimanjaro National Park. Reprinted from World Imagery Basemap (Esri, Maxar, Earthstar Geographics, and the GIS User Community) under a CC BY license, with permission from Esri Deutschland GmbH, original copyright 2024.

Weather stations, mainly for measuring precipitation, were already established in Tanzania in colonial times [11,12], but almost exclusively in the populated areas below the forest belts. Within the framework of several ecological projects of the German Research Foundation DFG, we were able to establish a network of weather stations on Kilimanjaro, Meru, the North Pare, the South Pare, West Usambara, East Usambara and Nguru (Figs 1 and 2) from 1996 onwards. The extensive climate network on Kilimanjaro, which extends from 700 to 4600m (Fig 3), was later partially integrated into the DFG research units KiLi (“Kilimanjaro under global change”) and later Kili-SES (“The role of nature for human well-being in the Kilimanjaro Social-Ecological System”), the latter project still ongoing. It includes 75 rainfall stations (Fig 3, S1 Table), where we mainly measure temperature, humidity, partly also wind speed and fog and in addition 33 stations, where we only measure temperature and humidity. For this study we have also included available data from the Tanzania Meteorological Agency, private companies and church missions from elevations below 1700 m (Fig 3, S1 Table), thus extending our own 75 rainfall stations to 125. We established the 21 stations on the other mountains after 2010 and complemented them with 26 stations of other sources (S1 Table), ranging from 120 to 2450m. Precipitation is one of the most fundamental meteorological elements and a highly dynamic one that changes its intensity, frequency and duration under the influence of topographic parameters such as elevation, vegetation and land use and is therefore more difficult to predict than temperature [13]. Therefore, and since the available dataset on precipitation is more extensive than that on other parameters, we here focus on mean annual precipitation (MAP).

## Materials and methods

### Study sites

The mountains studied lie on a transect that extends 350 km inland from the Tanzanian coast. Fourteen of the sixteen peaks studied belong to the Eastern Arc, a chain of ancient crystalline mountains in south-eastern Kenya and eastern Tanzania: two peaks are part of the North Pare (2200 m), three peaks belong to the South Pare (2450 m). The other seven peaks of the Eastern Arc are on West Usambara (2200 m), East Usambara (1400 m) and Nguru (2400 m). The other two mountains are huge volcanoes: Meru, at around 4600m the second highest mountain in Tanzania, and Kilimanjaro at 5900 the highest mountain in Africa (Figs 1 and 2, S1 Table).

Along its huge elevational gradient, Kilimanjaro covers several important natural ecosystems of tropical Africa, from lowland savannah to montane forest belts and alpine ecosystems (Fig 2). The natural habitats below 1800 m are greatly reduced due to agriculture and fodder production, and these areas are more or less densely populated [15]. For a detailed description of the most important vegetation habitats at Kilimanjaro, see [16,17]. The situation on Meru is similar, while the other mountains do not reach into the alpine climate zone. Only the highest peaks of the Eastern Arc Mountains are still covered by montane forest remnants, while the lower slopes are mostly cultivated and surrounded by agriculture or savannah vegetation at the foothills.

The climate on Kilimanjaro, located 300 km south of the equator, is typically tropical-equatorial and depends on the elevation. The mean annual temperature decreases linearly from 24°C in the savannah at 700 m a.s.l. to -7°C at the summit, while annual precipitation has a hump-shaped pattern, peaking at mid-altitudes in the montane forest belt. Due to wind exposure from the Indian Ocean, the southern and eastern slopes are wetter than the northern and western slopes [14,17]. This is similar on Meru, while on the other mountains, which are much lower, MAP rises to the summit according to our measurements.

### Experimental design

Precipitation data were collected with cumulative gauges and in parallel with automatic single tipping buckets connected to a data logger. In general, we used the data from the cumulative gauges, as this type has proven to be more reliable and has fewer failures than the automatic gauges. Although no objective quality correction could be applied, the use of accumulated rainfall should generally minimize errors and data inhomogeneity [13]. In case of missing data, the gaps were filled by data from the automatic gauges. Details of these stations (serial number of each station (if available), name of the station, start and end dates of the precipitation record) and elevation are given in S1 Table. On Kilimanjaro we have selected only stations with at least 10 years of data (without counting gaps in records), with the exception of 15 of our own stations in the montane and alpine zones with at least 5 years, where records are sparse. On the other mountains, where we started our measurements after 2010, most own stations have at least 5 years of data. The period of records at the stations varied. In the lower areas records started mainly in the middle of the last century, our own records (mainly in the upper areas) started at the end of the last century (S1 Table). WC provides data from 1950–2000, and C from 1979–2013 [10]. We have accepted these discrepancies in the recording periods in order to have a sufficiently large data set for such a comparison, and because precipitation changes have been marginal at Kilimanjaro in recent decades [18].

For precipitation measurements, we used cumulative gauges consisting of a funnel, a pipe and a canister, sealed with silicon, that are read manually about every three months. In addition, we measured precipitation with Pronamic single spoon tipping buckets at 0.2mm resolution connected to a Driesen & Kern DK311 ruggedPlus MultiLog Datalogger. On plots outside the forest belt, these gauges are installed at least 50 cm above the canopy to prevent back-splash. Inside the montane forest zone of Kilimanjaro, we placed the gauges in clearings (see [13]).

### Statistical analysis

Following the procedure of Ref. [2] we calculated the percent bias (pbias) that reflects the average tendency of the modelled precipitation values p_sim_ to be larger or smaller than their observed values P_obs_ at the stations. The optimal value of pbias is 0, with low values indicating accurate model simulation. Since we are only interested in overall bias disregarding over- or underestimation, we use the absolute numbers. Pbias is defined as follows:

$$pbias=100*\left(\frac{\sum_{i=0}^{n}\left|p_{{sim}_{i}}-p_{obs_{i}}\right|}{\sum_{i=0}^{n}p_{obs_{i}}}\right)$$

Additionally, we also report the mean absolute error (mae) which is defined as:

$$mae=\frac{1}{n}\left(\sum_{i=0}^{n}\left|p_{{sim}_{i}}-p_{obs_{i}}\right|\right)$$

Validation between observed and modelled rainfall data was performed with Pearson’s correlation coefficient. Extraction of WC and C data was done with ArcMap 10.8.2

To validate the performance of WC and C, we compared it to our own data using the highest resolution of the WC and C data (30 arc seconds), which has a better performance than the courser resolution of 0.25° gridded data [2]. For this, we calculated the mean absolute error (mae) and the percentage bias (pbias). We performed this calculation separately for the dry north slope and the wet south slope, which have different precipitation regimes. This separation of the ecoclimatic regions shown in Fig 3 was in accordance with [14].

## Results

Table 1 shows the calculated errors of the global climate data for all mountains studied. Performance is quite poor with a mean absolute error of 421 mm for WC and 407 mm for C. However, the mean deviation of modelled and measured maximum MAP is drastic with 926 mm for WC and 825 mm for C. Performance is generally better in the foothills than on the mountain slopes.

**Table 1:** Metrics for a comparison between WorldClim (WC) and CHELSA (C) data and the measured data of mean annual precipitation (MAP) based on 170 ground stations.

	Meru (n = 4)	Kili (n = 123)	NP (n = 14)	SP (n = 9)	WU (n = 4)	EU (n = 13)	Ngu (n = 3)	Foot (n = 15)	Mount (n = 32)
mae WC (mm)	643	467	159	237	240	358	554	155	372
pbias WC (%)	36	28	17	24	18	21	34	17	25
Deviation of max MAP WC (mm)	1182	1949	475	574	503	942	854		1182
mae C (mm)	318	467	265	265	153	190	421	119	310
pbias C (%)	17	31	24	25	13	11	23	14	22
Deviation of max MAP C (mm)	720	1811	658	680	374	777	757		777

mae = mean absolute error, pbias = percentage bias, Kili = Kilimanjaro, NP = North Pare, SP = South Pare, WU = West Usambara, EU = East Usambara, Ngu = Nguru, Foot = Foothills without Kilimanjaro, Mount = Mountain areas without Kilimanjaro. As the maximum MAP only occurs in the higher mountain areas, no value is given for the foothills.

**Fig 4:**
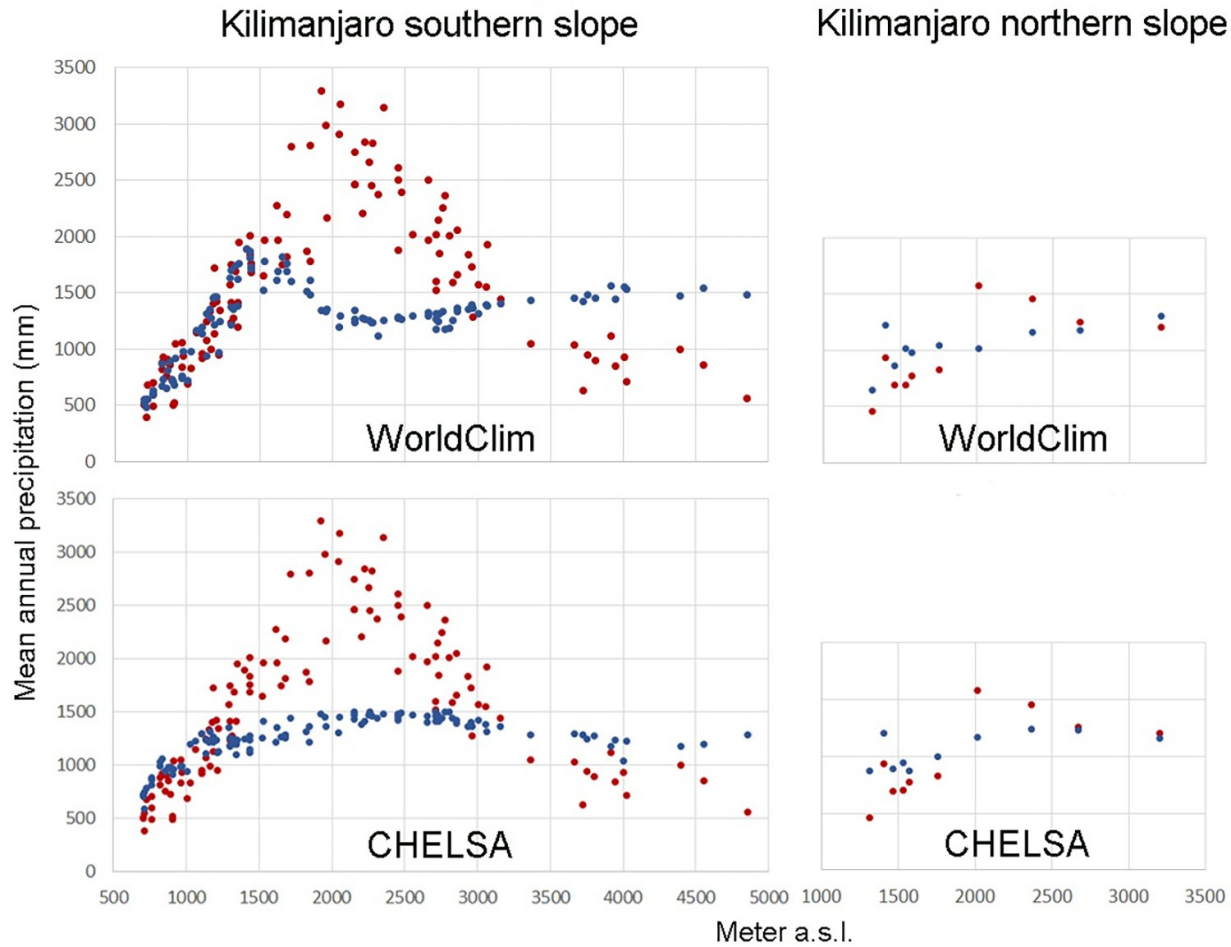
Comparison of measured MAP (red dots) and modelled values of WC and C (blue dots) for the southern and northern slope of Kilimanjaro separately.

Fig 4 and Table 2 show the results of these comparisons for Kilimanjaro, where most of the data were available. On the southern slope, where most of the data come from (Fig 3), WC and C perform quite well in the lower ranges up to about 1700 m (in the case of WC) and about 1300 m (in the case of C), with a mean absolute error of 125 mm and 202 mm.

**Table 2:** Metrics for a comparison between WorldClim (WC) and CHELSA (C) data and the measured data of mean annual precipitation (MAP) based on 125 ground stations on Kilimanjaro.

	Lower southern slope	Higher southern slope	Northern slope
mae WC (mm)	125	855	243
pbias WC (%)	12	46	26
mae C (mm)	202	693	210
pbias C (%)	25	35	26
Pearson’s r WC	0.94***	-0.56***	0.64*
Pearson’s r C	0.81***	0.62***	0.85**
max. MAP measured (mm)	-	**3.299**	**1582**
max. MAP WC (mm)	-	1.893	1313
max. MAP C (mm)	-	1.508	1242
Elevation max. MAP (m a.s.l.)	-	**1920**	**2010**
Elevation max. MAP WC (m a.s.l.)	-	1400	3200
Elevation max. MAP C (m a.s.l.)	-	2770	2360

mae = mean absolute error, pbias = percentage bias, r = Pearson correlation coefficient, *** p<0.001, ** p<0.01, *p<0.05. As the maximum MAP only occurs in the higher mountain areas, no value is given for the lower southern slope.

Further up, however, the agreement between measured and modelled data becomes very poor. C shows at least a moderate correlation, but the mean absolute error is with 702 mm very high. The correlation of C is even negative, showing an opposite trend of precipitation amount with altitude compared to the measured data (r = - 0.54) and an absolute error of 859 mm. This poor performance is also reflected in the amount and elevation of the maximum values: the maximum measured MAP was 3299 mm at 1920 m a.s.l., the maximum modelled MAP of WC was 1893 mm at 1400 m a.s.l. and of C 1508 mm at 2770 m a.s.l.

On the northern slope, the correlation was better; however, the maximum of measured MAP was 1582 mm at 2010 m a.s.l., while the maximum of modelled MAP of WC was 1313 mm at 3200 m a.s.l. and was 1242 mm at 2360 m a.s.l. in case of C (Table 1).

## Discussion

Both databases perform quite well in low-lying areas where a comparatively dense network of weather stations already existed. However, within the ecologically most important vegetation zones (in terms of biodiversity and ecosystem services, especially rainwater input), performance is poor and drastic discrepancies exist. Not only is the maximum MAP severely underestimated (e.g. on the southern slope of Kilimanjaro by about 50% by both models), but the rainfall trends were also completely unrealistic with deviations in the elevation of the rainfall maximum by up to 850 elevation metres. Comparing WC and C, C performs slightly better in most cases. But also the deviations of C are partly drastic, e.g. the mean absolute error of 700 mm along the higher part of the southern slope of Kilimanjaro and the difference between measured and modelled maximum MAP of 1791 mm.

Obviously, there is a slight trend towards greater deviations with increasing height (and thus area) of the mountains studied, which could be related to interpolation errors due to the larger area without station data.

Lower correlation with observed precipitation over complex (tropical) mountainous regions was already found by [7] for CW and by [19] for satellite-based precipitation estimates in two areas in Tanzania. [2] also found the lowest performance of downscaled grid-based rainfall data in the tropics, attributing this to the poor quality of the station data. Although surface datasets are not necessarily the ’truth’ as many factors influence the true value (exploitation of data, measurement equipment), inconsistencies or errors can be reduced through careful selection of data and quality control [7]. For our data, we can therefore exclude this explanation. Based on such data, more meaningful models can be built, as has been done for Kilimanjaro for precipitation [17] (Hemp, 2006) and for precipitation, temperature and humidity [13] with a much higher resolution.

The fact that the measured periods sometimes deviate considerably from the modelled data is hardly significant: firstly, the longest records and the highest deviations in the measurement periods occurred in the lowlands, where the models performed best, while at higher elevations, with the much higher agreement in the recording periods, the deviation in the amount of precipitation values was greatest. Furthermore, precipitation changes have not been very pronounced in recent decades [18].

The observed discrepancies are thus more related to the downscaling approach used, to the lack of available data for the model and to the grid cell size of 1 km, which is much too large in complex terrain. In the case of C, where data from a downscaled satellite-gauge reanalysis (ERA5) is merged with further remote sensing data, possible sources of error are even more difficult to detect.

One aspect that has not been addressed in this study is the impact of water input from fog. On Kilimanjaro, this input is of less importance due to the weak and moderate wind speeds [20] (Hemp, 2005b). In the Pare Mountains, however, this input is important. On Mwala, a peak of the South Pare, for example, water input from fog can be more than twice the rainfall, increasing the MAP from 740 mm to 2303 mm, creating cloud forest habitats with unique vegetation [21,22], the existence of which could not be explained by rainfall data alone. Taking this into account, the bias of the modelled data would have been greater.

We are only addressing the mean annual precipitation, the basic bioclimatic variable BIO12 [10]. However, we can assume that the other precipitation-related bioclimatic variables BIO13-BIO19 modelled by WC and C have a similar deviation on Kilimanjaro and very likely also on other tropical mountains.

## Conclusions

**Fig 5:**
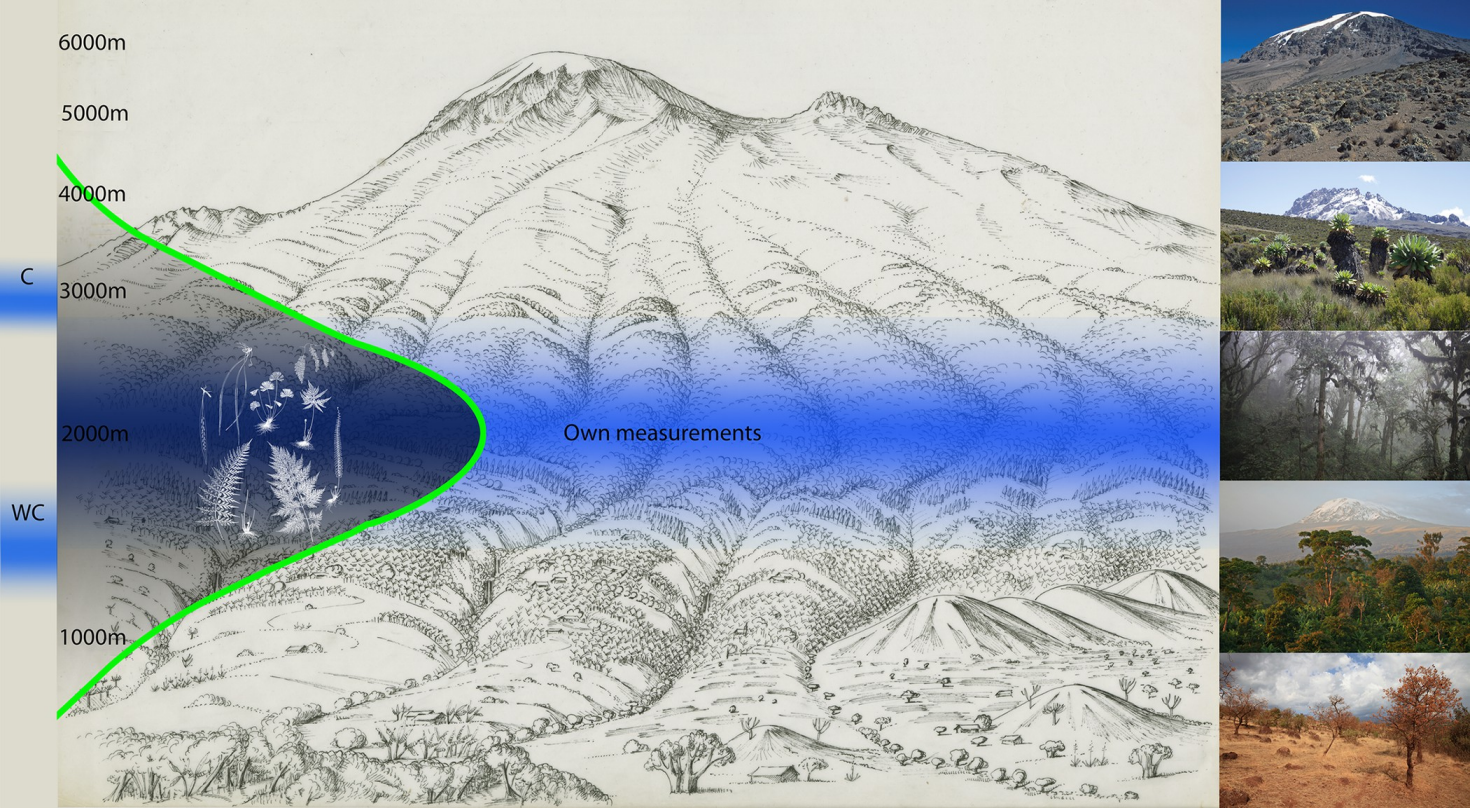
Comparison of measured and modelled MAP maxima in relation to biodiversity patterns on the southern slope of Kilimanjaro. MAP data of WC and C cannot explain biodiversity patterns (e.g. of pteridophytes, green curve, from [25] and vegetation zonation (right images; from bottom to top: Colline savannah zone, submontane agroforestry zone, montane forest zone, subalpine and alpine zone) due to their incorrect rainfall maxima (blue zones).

In areas of the world with very good coverage by weather stations (e.g. North America, Western Europe), the accuracy of the interpolated climate databases is correspondingly high in most places. For all those parts of the world where there are no such stations, the currently available data sets will continue to be used extensively despite their disadvantages. The promising results in predicting extreme precipitation using a neural network [23] could point the way to modelling grid-based precipitation data. Until this is possible, existing "high-resolution" databases in tropical regions must be used with caution and conclusions must take into account the risk of error, which can be drastic, as shown on Kilimanjaro. Tools such as WC and C must be treated as what they are, at least in the tropical mountains: possible models that deviate more or less from reality. If such reanalysis products projecting into the past have such drawbacks, their failure in future projections is likely to be even greater and would require more data for better results [24]. It is clear from this study that the conclusions of our research on biodiversity patterns and ecosystem functioning at Kilimanjaro [25,26] would have been significantly different if we had used global climate datasets instead of our own measurements (Fig 5). However, to obtain comprehensive data for the models, continuous long-term observations must be carried out, and this depends on the availability of long-term funding [27].

## Supporting information

S1 Table Details of the stations used. TMA: Tanzania Meteorological Authority.
(PDF)

## References

[1] Hijmans RJ, Condori B, Carillo R, Kropff MJ. A quantitative and constraint-specific method to assess the potential impact of new agricultural technology: the case of frost resistant potato for the Altiplano (Peru and Bolivia). Agricult Syst 2003;76: 895–911.

[2] Karger DN, Wilson AM, Mahony C, Zimmermann NE, Jetz W. Global daily 1 km land surface precipitation based on cloud cover-informed downscaling. Sci Data 2021;8: 307. https://www.nature.com/articles/s41597-021-01084-6. 34836980 10.1038/s41597-021-01084-6 PMC8626457

[3] Menne MJ, Durre I, Korzeniewski B, McNeill S, Thomas K, Yin X et al. Global Historical Climatology Network—Daily (GHCN-Daily), Version 3. NOAA National Climatic Data Center. doi: 10.7289/V5D21VHZ (2012) (accessed 31 July 2023).

[4] Dieulin C, Mahé G, Paturel J-E, Ejjiyar S, Tramblay Y, Rouché N, EL Mansouri B. A New 60-year 1940/1999 Monthly-Gridded Rainfall Data Set for Africa. Water 2019;11: 387.

[5] Harris I, Osborn TJ, Jones P, Lister D. Version 4 of the CRU TS monthly high-resolution gridded multivariate climate dataset. Sci Data 2020;7: 1–18. 32246091 10.1038/s41597-020-0453-3 PMC7125108

[6] Hersbach H, de Rosnay P, Bell B, Schepers D, Simmons A, Soci C et al. Operational global reanalysis: progress, future directions and synergies with NWP. ERA Report Series 2018. doi: 10.21957/tkic6g3wm

[7] Hijmans RJ, Cameron SE, Parra JL, Jones PG, Jarvis A.Very high resolution interpolated climate surfaces for global land areas. Int J Climatol 2005;25: 1965–1978.

[8] Liu Z, Liu Y, Wang S, Yang X, Wang L, Baig MHA, Chi W, Wang Z. Evaluation of spatial and temporal performances of ERA-Interim precipitation and temperature in mainland China. J Clim 2018;31: 4347–4365.

[9] Fick SE, Hijmans RJ. WorldClim 2: new 1-km spatial resolution climate surfaces for global land areas. Int J Climatol 2017;37: 4302–4315.

[10] Karger DN, Brun PB, Zimmermann E. Climatologies at high resolution for the earth’s land surface areas. EnviDat 2021b. 10.16904/envidat.228.v2.1. PMC5584396 28872642

[11] Volkens G., 1897. Der Kilimandscharo. Darstellung der allgemeineren Ergebnisse eines fünfzehnmonatigen Aufenthalts im Dschaggalande. Berlin: Reimer; 1897.

[12] Widenmann A. Die Kilimandscharo-Bevölkerung. Anthropologisches und Ethnographisches aus dem Dschaggalande. Petermanns geographische Mitteilungen, Ergänzungs-Heft 1899;129: 1–105.

[13] Appelhans T, Mwangomo E, Otte I, Detsch F, Nauss T, Hemp A. Eco-meteorological characteristics of the southern slopes of Kilimanjaro, Tanzania. Int J Climatol 2016;36: 3245–3258.

[14] Hemp A. Continuum or zonation? Altitudinal gradients in the forest vegetation of Mt. Kilimanjaro. Plant Ecol 2005a;84(1): 27–42.

[15] Hemp A, Hemp C. Broken bridges. The isolation of Kilimanjaro’s ecosystem. Glob Change Biol 2018. doi: 10.1111/gcb.14078 29504230

[16] Hemp A. Ecology of the pteridophytes on the southern slopes of Mt. Kilimanjaro. Part II: Habitat selection. Plant Biol 2001;3: 493–523.

[17] Hemp A. Vegetation of Kilimanjaro: hidden endemics and missing bamboo. Afr J Ecol 2006;44: 305–328. doi: 10.1111/j.1365-2028.2006.00679.x

[18] Otte I, Detsch F, Mwangomo E, Hemp A, Appelhans T, Nauss T. Multidecadal Trends and Interannual Variability of Rainfall as Observed from Five Lowland Stations at Mt. Kilimanjaro, Tanzania. J. Hydrometeor. 2017;18: 349–361.

[19] Mashingia F, Mtalo F, Bruen M. Validation of remotely sensed rainfall over major climatic regions in Northeast Tanzania. Phys Chem Earth 2014;67–69: 55–63.

[20] Hemp A. Climate change driven forest fires marginalizes the ice cap wasting on Mt. Kilimanjaro. Glob Change Biol 2005b;11: 1013–1023.

[21] Cribb PJ, Hemp A. Rhipidoglossum pareense (Orchidaceae: Epidendroideae), a new species from Tanzania. Kew Bull 2022. doi: 10.1007/S12225-022-10027-2

[22] Darbyshire I, Hemp A. A further new species of Isoglossa (Acanthaceae) from the Eastern Arc Mountains of Tanzania. Kew Bull 2022; 78: 499–507. doi: 10.1007/s12225-023-10103-1

[23] Zhang Y, Long M, Chen K, Xing L, Jin R, Jordan MI, Wang J. Skilful nowcasting of extreme precipitation with NowcastNet. Nature 2023;619: 526–532. doi: 10.1038/s41586-023-06184-4 37407824 PMC10356617

[24] Björklund J, Seftigen K, Stoffel M, Fonti MV, Kottlow S, David C. Frank DC et al. Fennoscandian tree-ring anatomy shows a warmer modern than medieval climate. Nature 2023;620: 97–103. doi: 10.1038/s41586-023-06176-4 37532816

[25] Peters MK, Hemp A, Appelhans T, Behler C, Classen A, Detsch F, et al. Predictors of elevational biodiversity gradients change from single taxa to the multi-taxa community level. Nat Commun 2016;7: 13736. doi: 10.1038/ncomms13736 28004657 PMC5192166

[26] Peters MK, Hemp A, Appelhans T, Becker JN, Behler C, Classen A et al. Climate-land-use interactions shape tropical mountain biodiversity and ecosystem functions. Nature 2019;568: 88–92. doi: 10.1038/s41586-019-1048-z 30918402

[27] Editorial. We must get a grip on forest science—before it’s too late. Nature 2022;608: 449. doi: 10.1038/d41586-022-02182-0 35974155

