# Supplementary material for: Weather or not—Global climate databases: Reliable on tropical mountains?
Source: PLoS One. 2024 Mar 13;19(3):e0299363. doi: 10.1371/journal.pone.0299363 (PMC10936801; doi:10.1371/journal.pone.0299363)
Supplement: S1 Table — TMA: Tanzania Meteorological Authority. (PDF) [file pone.0299363.s001.pdf]

S1 Table. Details of the stations used.

Name and serial number (if available)	Peaks and mountain ranges	Source	Record lenght (years)	Record period
MERU_Ngurdoto_Ranger	Meru	own data	6	2015-2023
MERU_1710	Meru	own data	9	2011-2023
MERU_2130	Meru	own data	9	2013-2023
MERU_2510	Meru	own data	8	2013-2023
MAI2	Kilimanjaro	own data	10	2013-2023
MAI3	Kilimanjaro	own data	10	2012-2012
MAI4	Kilimanjaro	own data	10	2013-2023
MAI5	Kilimanjaro	own data	10	2012-2012
Chala	Kilimanjaro	own data	16	2006-2023
FED3	Kilimanjaro	own data	8	2014-2023
FED4	Kilimanjaro	own data	8	2014-2023
FED5	Kilimanjaro	own data	10	2013-2023
FER0	Kilimanjaro	own data	10	2012-2023
FLM1	Kilimanjaro	own data	10	2012-2023
FLM3	Kilimanjaro	own data	10	2011-2023
FLM6	Kilimanjaro	own data	11	2012-2023
FOC1	Kilimanjaro	own data	22	2000-2023
FOC2	Kilimanjaro	own data	10	2012-2023
FOC4	Kilimanjaro	own data	11	2012-2023
FOC5	Kilimanjaro	own data	22	2001-2023
FOD1	Kilimanjaro	own data	10	2013-2023
FOD2	Kilimanjaro	own data	16	2006-2023
FOD3	Kilimanjaro	own data	11	2012-2023
FPD1	Kilimanjaro	own data	11	2012-2023
FPD2	Kilimanjaro	own data	17	2001-2023
FPD4	Kilimanjaro	own data	11	2012-2023
FPD5	Kilimanjaro	own data	10	2013-2023
FPO1	Kilimanjaro	own data	10	2013-2023
FPO4	Kilimanjaro	own data	11	2012-2023
FPO5	Kilimanjaro	own data	6	2013-2019
GRA4	Kilimanjaro	own data	11	2012-2023
GRA5	Kilimanjaro	own data	10	2012-2023
HEL4	Kilimanjaro	own data	8	2014-2023
HEL5	Kilimanjaro	own data	11	2012-2023
HOM3	Kilimanjaro	own data	10	2012-2023
Horombo	Kilimanjaro	[28]; own data	23	1945-1958; 2010-2023
Kidia 1800	Kilimanjaro	own data	19	2001-2023
Kidia 2400	Kilimanjaro	own data	14	2002-2023
Kidia 2700	Kilimanjaro	own data	6	2002-2023
Kidia 3000 isale	Kilimanjaro	own data	16	2003-2023
Kidia scientific station	Kilimanjaro	own data	25	1997-2023
Kidia Turm	Kilimanjaro	own data	21	2001-2023
Kidia_olotu	Kilimanjaro	own data	10	2002-2019
Lerangwa_ranger	Kilimanjaro	own data	6	2011-2023
Machame 2400	Kilimanjaro	own data	21	2001-2023
Machame 2700	Kilimanjaro	own data	18	2002-2023
Machame 2900	Kilimanjaro	own data	17	2002-2023
Mandara hut	Kilimanjaro	own data	7	2015-2023
Marangu_2300	Kilimanjaro	own data	13	2007-2023
Masoka	Kilimanjaro	own data	18	2001-2023
Maua_rangerpost	Kilimanjaro	own data	11	2010-2023
Mweka	Kilimanjaro	own data	18	2001-2023
Mweka 2200	Kilimanjaro	own data	21	2001-2023
Mweka 3300	Kilimanjaro	own data	18	2001-2023
Mweka 4000	Kilimanjaro	own data	12	2002-2018
NALEMOR RANG R	Kilimanjaro	own data	6	2016-2023
NALEMORU	Kilimanjaro	own data	6	2016-2023
Nkweseko_station	Kilimanjaro	own data	14	2006-2023
Pius	Kilimanjaro	own data	12	2006-2023
Rongai_newgate	Kilimanjaro	own data	6	2016 -2023
SAV1	Kilimanjaro	own data	10	2012-2023
SAV3	Kilimanjaro	own data	10	2012-2023
SIMBA CAMP	Kilimanjaro	own data	5	2016 -2023
Weruweru 1900	Kilimanjaro	own data	11	2005-2023
Weruweru 2000	Kilimanjaro	own data	10	2005-2023
Weruweru 2100	Kilimanjaro	own data	11	2005-2023

Weruweru 2200	Kilimanjaro	own data	10	2005-2023
Weruweru 2300	Kilimanjaro	own data	11	2005-2023
Weruweru 2400	Kilimanjaro	own data	13	2005-2023
Weruweru 2600	Kilimanjaro	own data	11	2005-2023
Weruweru 2700	Kilimanjaro	own data	11	2005-2023
Weruweru 2800	Kilimanjaro	own data	11	2005-2023
Weruweru 2900	Kilimanjaro	own data	7	2005-2023
Weruweru 3000	Kilimanjaro	own data	13	2005-2023
Weruweru 3100	Kilimanjaro	own data	11	2005-2023
Weruweru 3200	Kilimanjaro	own data	5	2005-2013
Werweru 2500	Kilimanjaro	own data	13	2005-2023
93.37107 Lambo Sisal Estate; MAI0	Kilimanjaro	TMA; own data; TI	34	1966-1996; 2017-2023
9337131 TPRI	Kilimanjaro	TMA; own data; TI	26	1972-1989; 2013-2023
93.37064 Old Moshi Nursery, Kidia Rar	Kilimanjaro	TMA; own data; TI	69	1948-1991; 2014-2023
Kibohut	Kilimanjaro	[28]	13	1945-1958
Londorossi2_Hel1	Kilimanjaro	[28]	7	1947-1953
Huruma town	Kilimanjaro	private	26	1976-2001
KIA	Kilimanjaro	private	44	1972-2014
Kibo estate	Kilimanjaro	private	11	1999-2009
Kibonoto	Kilimanjaro	private	69	1931-2013
Kifufu estate	Kilimanjaro	private	46	1949-1990; 1997-2001
Kikafu estate	Kilimanjaro	private	40	1930-1962; 2003-2009
Kilema chini	Kilimanjaro	private	44	1942-2014
Kilema foreststation	Kilimanjaro	private	10	1993-2002
Kiyungi Sisal estate	Kilimanjaro	private	66	1935-2001
MACHARE estate	Kilimanjaro	private	25	1998-2022
Makoa_R	Kilimanjaro	private	14	2001-2014
Malilo (Prim. School)	Kilimanjaro	private	16	1987-2002
Namuai estate	Kilimanjaro	private	8	1986-1994
Old Moshi Shia (Prim. School)	Kilimanjaro	private	23	1980-2002
TPC Factory	Kilimanjaro	private	41	1974-2015
Tschibo 1d chombo	Kilimanjaro	private	20	2001-2022
Tschibo 2e gomberi=9337018 Singa C	Kilimanjaro	private	54	1933-1967; 2001-2022
Tschibo 3 f kaity	Kilimanjaro	private	20	2001-2022
Tschibo 4b kichomi	Kilimanjaro	private	20	2001-2022
Tschibo 5h kifumbu	Kilimanjaro	private	20	2001-2022
Tschibo 6g kilimanjaro	Kilimanjaro	private	20	2001-2022
Tschibo 7c mawingo	Kilimanjaro	private	20	2001-2022
Tschibo 8a tschibo	Kilimanjaro	private	20	2001-2022
URU estate	Kilimanjaro	private	22	2001-2022
Uru-West	Kilimanjaro	private	14	1989-2002
3337121 Osaki Forest	Kilimanjaro	TMA	20	1972-1993
9237006 Rongai_foreststation	Kilimanjaro	TMA	61	1931-1992
93.37004 Moshi Meteorological Statio	Kilimanjaro	TMA	111	1901-2014
93.37005 Kibosho Mission;	Kilimanjaro	TMA	105	1912-2014
93.37006 Rombo Mission;	Kilimanjaro	TMA	75	1930-2005
93.37015 Kilema Mission;	Kilimanjaro	TMA	93	1911-2004
93.37021 Lyamungu;	Kilimanjaro	TMA	66	1935-2001
93.37024 Kikuletwa Railway Station;	Kilimanjaro	TMA	35	1935-1970
93.37028 Arusha Chini Estate (TPC Lan	Kilimanjaro	TMA	63	1938-2001
93.37031 Himo Sisal Estate;	Kilimanjaro	TMA	61	1938-1999
93.37046 Marangu college	Kilimanjaro	TMA	31	1942-1995
93.37047 Friesian Farm;	Kilimanjaro	TMA	49	1941-1992
93.37068 Machame School	Kilimanjaro	TMA	18	1949-1964; 2007-2008
93.37069 Kikuletwa Hydro Station;	Kilimanjaro	TMA	18	1961-1979
93.37077 Ngare Nairobi;	Kilimanjaro	TMA	40	1953-2011
93.37082 Marakatembo;	Kilimanjaro	TMA	10	1961-1974
93.37083 Matadi Estate;	Kilimanjaro	TMA	19	1961-1989
93.37091 W.D. & I.D. Moshi;	Kilimanjaro	TMA	60	1955-2014
9337036 Kahe Railwaystation	Kilimanjaro	TMA	63	1936-2006
9337072 Kifaru Sisal estate	Kilimanjaro	TMA	34	1951-1985
9337105 TPC Karanga	Kilimanjaro	TMA	23	1966-1989
9337123 Maua Seminary	Kilimanjaro	TMA	31	1973-2003
Kifaru School	North Pare, Kifaru	own data	8	2015-2023
Kifaru 1700	North Pare, Kifaru	own data	7	2015-2023
Kindoroko 1700	North Pare, Kindoroko	own data	11	2011-2023
Kindoroko 2000	North Pare, Kindoroko	own data	13	2006-2023
Kiverenge 1000	North Pare, Kiverenge	own data	8	2015-2023
Kiverenge 1300	North Pare, Kiverenge	own data	7	2016-2023

Kiverenge 1700	North Pare, Kiverenge	own data	9	2012-2023
Goofi 1800	North Pare, Ngoofi	own data	6	2007-2023
9337090 Nymba Mungu	North Pare (lowlands)	TMA	31	1961-1991
9337023 Kisangiro Railway	North Pare (lowlands)	TMA	28	1935-1963
9337146 Mwanga agriculture	North Pare (lowlands)	TMA	29	1986-2014
9337033 Kisanagara Estate	North Pare (lowlands)	TMA	78	1934-2011
Shingatini Middle school	North Pare (uplands)	TMA	52	1950-2001
Kilomeni Mission	North Pare, Kindoroko	TMA	68	1928-1995
Kwizu	South Pare (lowlands)	own data	5	2018-2023
Mwala 1300	South Pare, Mwala	own data	5	2017-2023
Mwala 1700	South Pare, Mwala	own data	6	2017-2023
Shengena 900	South Pare, Shengena	own data	5	2010-2017
Shengena 2400	South Pare, Shengena	own data	5	2010-2017
Vumari hospital	South Pare, Vumari	own data	5	2015-2023
Vumari 1700	South Pare, Vumari	own data	5	2016-2023
Gonja	South Pare (lowlands)	TMA	45	1941-1986
Same	South Pare (lowlands)	TMA	65	1935-1999
Lutindi hospital	West Usambara, Lutindi	private	9	1989-2014
Mazumbai	West Usambara, Mazumbai	[29]	36	1945-1981
9438059_Mnazi Sisal Estate	West Usambara (lowlands)	TMA	25	1967-1992
9438001_Lwandai Mission School	West Usambara (uplands)	TMA	41	1931-1971
NILO CAMP	East Usambara, Nilo	own data	5	2018-2023
NILO_1500	East Usambara, Nilo	own data	5	2018-2023
9538054_Mjesani Sisal Estate	East Usambara (lowlands)	TMA	25	1966-1991
9438052_Bamba Sisal Estate	East Usambara (lowlands)	TMA	19	1966-1985
9538028_Muheza Sisal Estate	East Usambara (lowlands)	TMA	50	1949-1999
9438033_Lugongo Sisal Estate	East Usambara (lowlands)	TMA	30	1955-1985
9438054_Mwele Sisal Estate	East Usambara (lowlands)	TMA	45	1949-1994
9538063_Bombwera Sisal Estate	East Usambara (lowlands)	TMA	26	1967-1993
9438027_Daluni	East Usambara (lowlands)	TMA	27	1956-1983
9538041_Lunguza Forest	East Usambara (uplands)	TMA	32	1961-1993
9538003_Amani Malaria Unit	East Usambara, Amani	TMA	99	1901-1999
9538014_Kwamkoro Estate	East Usambara, Kwamkoro	TMA	23	1962-1985
9538075_Magroto Estate	East Usambara, Magoroto	TMA	19	1974-1993
Nguru_Maskati_Mission	Nguru	own data	5	2018-2023
Nguru_2100	Nguru	own data	5	2018-2023
Nguru_2300	Nguru	own data	5	2018-2023

TMA: Tanzania Meteorological Agency

References

28. Hedberg O. Features of afroalpine plant ecology. Acta Phytogeograph Suec 1964;49: 1-144.
29. Lundgren L, Lundgren B. Rainfall, interception and evaporation in the Mazumbai Forest Reserve, West Usambara Mts., Tanzania and their importance in the assessment of land potential. Geogr Ann Ser B 1979;61: 157-178.
